# Supplementary material for: Discovery and pharmacophoric characterization of chemokine network inhibitors using phage-display, saturation mutagenesis and computational modelling
Source: Nat Commun. 2023 Sep 16;14:5763. doi: 10.1038/s41467-023-41488-z (PMC10505172; doi:10.1038/s41467-023-41488-z)
Supplement: Supplementary file 4 — Source Data [file 41467_2023_41488_MOESM4_ESM.zip › SourceData/scripts/xNNKs_20230808183658.tar!/xNNKs/analysis/heatmaps/SV_HD2X_conservative_heatmap.pdf]

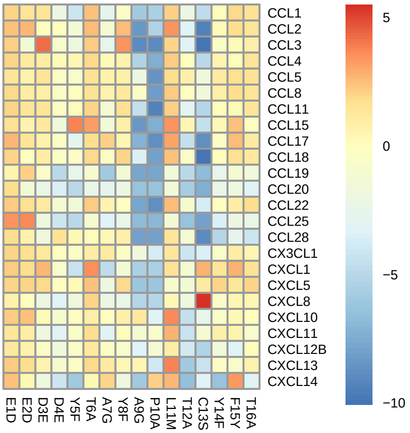

CCL1
CCL2
CCL3
CCL4
CCL5
CCL8
CCL11
CCL15
CCL17
CCL18
CCL19
CCL20
CCL22
CCL25
CCL28
CX3CL1
CXCL1
CXCL5
CXCL8
CXCL10
CXCL11
CXCL12B
CXCL13
CXCL14
E1D
E2D
D3E
D4E
Y5F
T6A
A7G
Y8F
A9G
P10A
L11M
T12A
C13S
Y14F
F15Y
T16A
5
0
−5
−10
